# Stability of Photo-Printed and Oil-Painted Irises in Ocular Prostheses Subjected to Weathering

**DOI:** 10.7759/cureus.109555

**Published:** 2026-05-24

**Authors:** James Dudley, Nafij Bin Jamayet, Chong K Xin, Jocelyn W Shi Kei, Naveen Chabra

**Affiliations:** 1 School of Dentistry, Adelaide University, Adelaide, AUS; 2 School of Dentistry, IMU University, Kuala Lumpur, MYS

**Keywords:** colour stability, dimensional stability, iris fabrication, ocular prosthesis, oil-painted, photo-printed

## Abstract

Introduction

Iris fabrication is central to ocular prosthesis aesthetics, yet comparative evidence on oil‑painting versus photo‑printing under environmental stressors remains limited. This study evaluated colour stability, dimensional stability and objective acceptability of conventionally oil‑painted and photo‑printed irises in ocular prostheses following exposure to weathering conditions.

Methods

Forty ocular prostheses were fabricated, comprising 20 oil‑painted and 20 photo‑printed irises. Ocular prostheses fabricated with each method were divided into two subgroups: 10 were subjected to outdoor weathering conditions for two weeks, and 10 were maintained in a dark room as controls, yielding four experimental groups: oil-painted weathered, oil-painted non-weathered, photo-printed weathered, and photo-printed non-weathered. Colour change was quantified using CIELAB colour difference formula values in Adobe Lightroom Classic (Adobe Inc., San Jose, California, USA), and iris size was assessed with Adobe Photoshop (Adobe Inc.). A researcher‑designed questionnaire compared the acceptability of oil-painted and digitally printed irises in ocular prostheses.

Results

Photo-printed non-weathered prostheses demonstrated the smallest mean colour value of all other groups. Following exposure to outdoor weathering conditions, colour changes in photo-printed irises were significantly larger than those in oil-painted irises (p<0.001). Photo-printed weathered irises were significantly smaller than oil-painted non-weathered irises (p=0.004), photo-printed non-weathered irises (p<0.001), and oil-painted weathered irises (p=0.002). In general, respondents rated oil-painted irises as more acceptable than photo-printed irises when compared with a natural human iris.

Conclusions

Oil-painted irises in ocular prostheses exhibited greater colour and dimensional stability compared with photo‑printed irises in ocular prostheses following exposure to outdoor weathering conditions. Oil‑painted irises were consistently judged more aesthetically acceptable than photo‑printed irises when compared with the natural human iris, underscoring their suitability for achieving durable and aesthetically pleasing prosthetic outcomes. Despite the efficiency and reproducibility offered by digital fabrication techniques, conventional oil‑painting methods currently remain the more reliable iris fabrication option for achieving durable and clinically acceptable aesthetic outcomes in ocular prostheses. Further research evaluating long‑term performance and advances in digital printing is warranted to enhance the viability of photo‑printed iris fabrication.

## Introduction

Ocular defects are among the most common extraoral maxillofacial deformities, classified as congenital or acquired. These defects can significantly affect facial aesthetics, visual function, and psychosocial well-being. Acquired causes often include trauma, retinoblastoma, and squamous cell carcinoma, which usually require surgical enucleation. Removal of the globe and surrounding periorbital tissues creates a defect that affects both anatomical support and facial aesthetics, making prosthetic rehabilitation necessary for restoring appearance and patient confidence [1,2]. To achieve an optimal aesthetic outcome, the prosthesis must match the dimensions of the defect precisely and replicate the ocular colour, particularly the iris, which is the central focus of ocular aesthetics. Conventional iris painting techniques need significant artistic skill and colour judgment. Outcomes also depend on the type of pigment used. Water and oil‑based media are commonly used; however, several studies have reported pigment dispersion into the scleral region during polymerization and mould closure, which can affect intrinsic characterization [3,4].

Digital techniques have been proposed to address these and other issues, involving using photographs and printing the contralateral iris for incorporation into the scleral shell. These methods provide advantages of speed and accuracy, although factors such as printing quality and bonding technique are critical determinants of success [5]. Environmental factors also affect prosthesis performance, with accelerated aging and colour degradation reported in tropical climates, highlighting the need to evaluate the durability of digitally printed irises under outdoor settings [3].

Despite these advances, there is limited evidence on how humans perceive the aesthetic outcomes of digitally printed ocular prostheses. The colour precision of photo‑printed irises compared with conventionally fabricated prostheses and natural eyes remains uncertain, as few comprehensive studies have evaluated their aesthetic outcomes. This study sought to determine the relative suitability of oil‑painted and photo‑printed irises in ocular prostheses following exposure to weathering conditions that simulate clinical prosthetic use.

The aim of this study was to evaluate the colour stability, dimensional stability and objective acceptability of conventionally oil‑painted versus photo‑printed irises in ocular prostheses following exposure to weathering conditions. The null hypotheses were: (1) There is no significant difference in colour stability or dimensional stability between ocular prostheses with conventionally oil‑painted irises and those with photo‑printed irises following exposure to weathering conditions. (2) There is no significant difference in objective acceptability ratings between ocular prostheses with conventional oil‑painted irises and those with photo‑printed irises following exposure to weathering conditions.

In this study, the term “objective acceptability” refers to instrument-based quantitative assessment of colour and dimensional stability, whereas “subjective acceptability” refers to perceptual evaluation obtained through questionnaire-based ratings of aesthetic outcomes.

## Materials and methods

Based on an a priori power analysis with an effect size of d=0.94, a significance level of α=0.05, and a statistical power of 1-β=0.95, the required total sample size was estimated as N=30 using G*Power (version 3.1.9.4; Heinrich Heine University Düsseldorf, Düsseldorf, Germany). An additional 15% of samples were considered for each test to counteract any handling errors. Therefore, a total of 40 samples were fabricated: 20 prostheses were fabricated using the conventional oil‑painting technique, and 20 were fabricated using the photo-printed iris technique.

The 40 custom‑made ocular prostheses were divided equally into two groups. Each group comprised 20 oil‑painted irises and 20 photo‑printed irises. Ten prostheses in each group were exposed to direct sunlight for three hours daily over a two‑week period, while 10 prostheses in each group were stored in a dark room. There were four experimental groups: (1) oil‑painted weathered (OPW), (2) oil‑painted non‑weathered (OPNW), (3) photo‑printed weathered (PPW) and (4) photo‑printed non‑weathered (PPNW).

**Figure 1:**
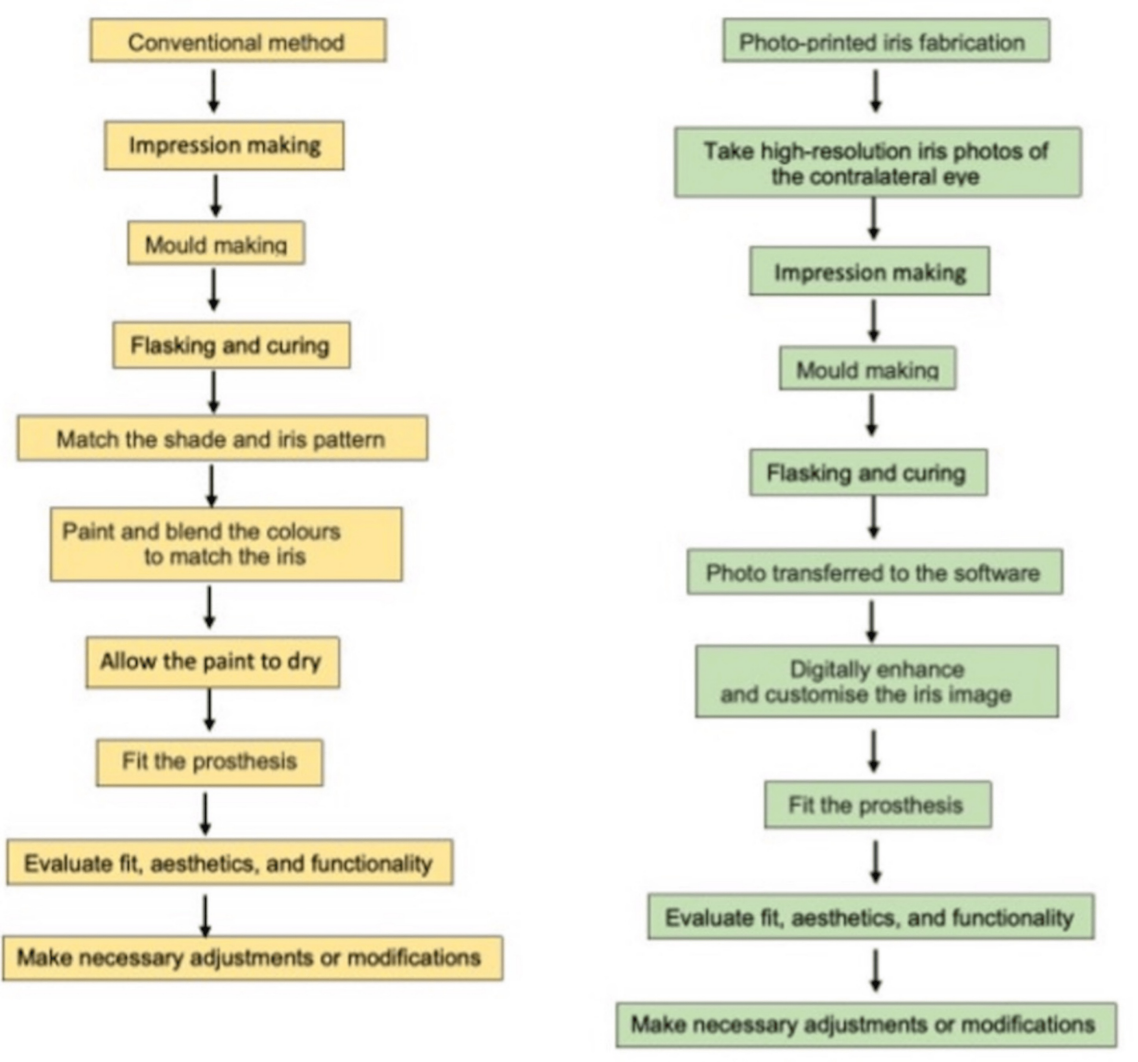
Flow chart of oil-painted and photo-printed iris fabrication

**Figure 2:**
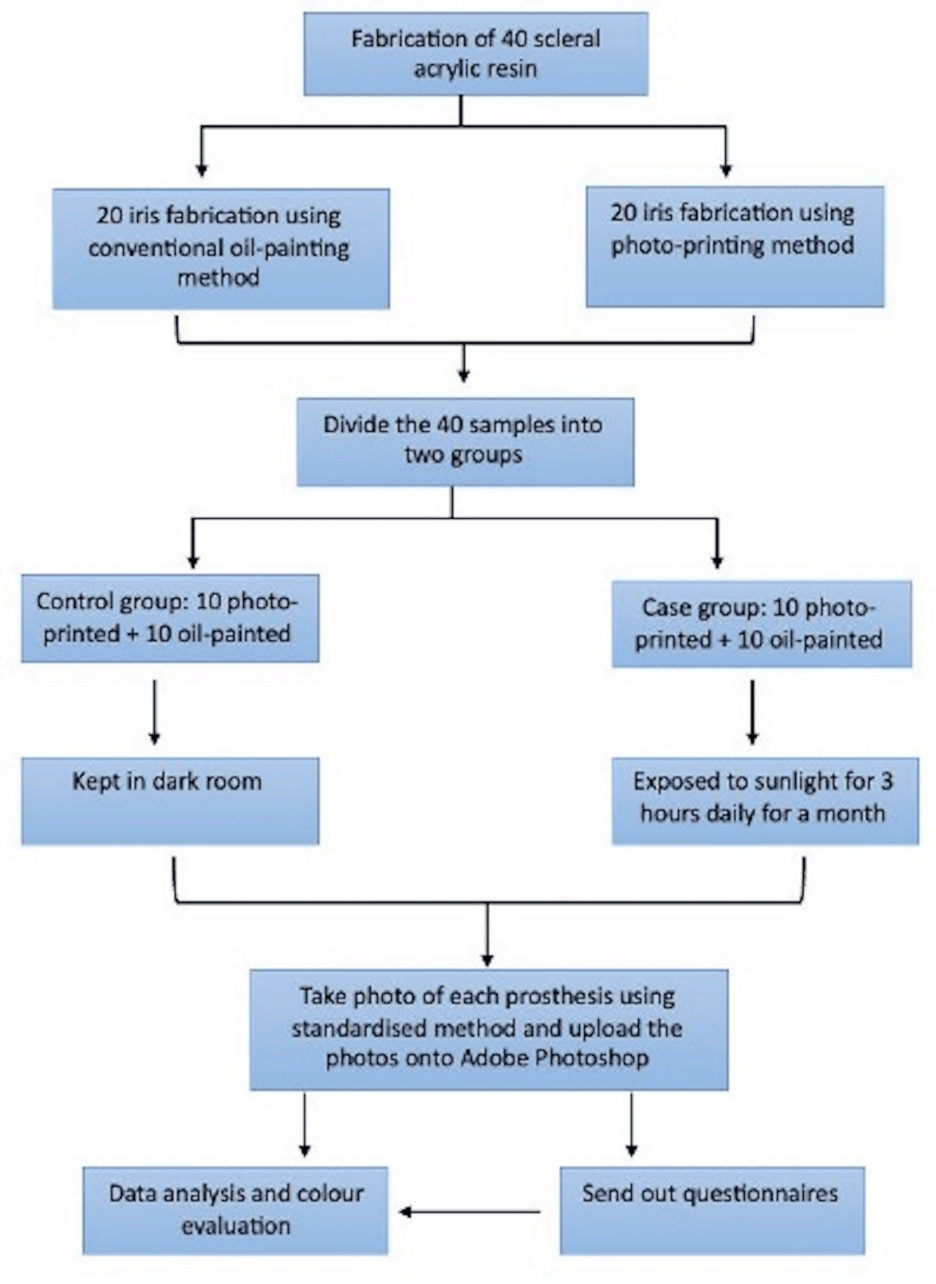
Study workflow

Two researchers received four weeks of training under the supervision of a specialist in ocular prostheses to ensure consistency in fabrication procedures. A previously prepared patient case mould was used for all prostheses in both groups to standardize size, dimensions, and volume. Iris coloration was standardized using a shade guide, with brown selected as the study colour. For the oil-painted group, irises were fabricated using brown oil paint. For the photo-printed group, a digital photograph of a natural brown human iris was captured and incorporated. Figure 1 presents the two iris fabrication workflows, and Figure 2 presents the overall study workflow.

**Figure 3:**
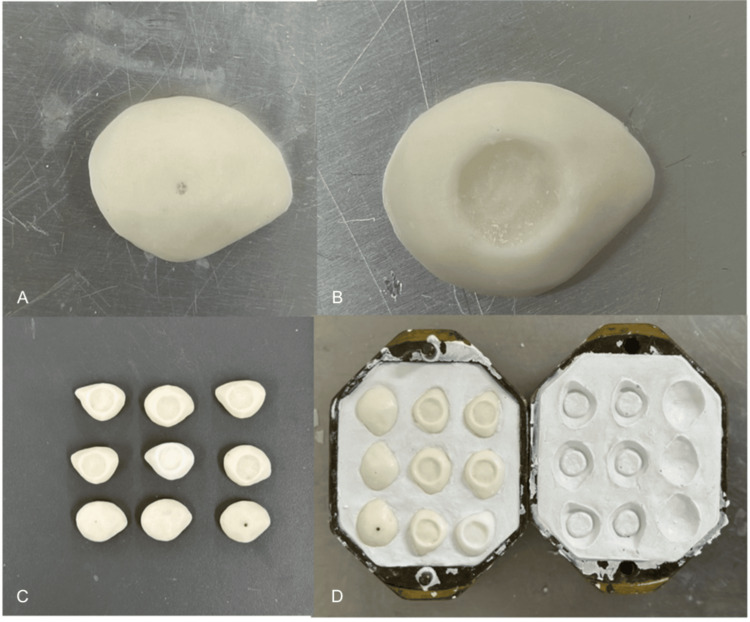
Custom-made ocular prosthesis fabrication: (A) centre of the sclera marked with a pencil; (B) depth of 3 mm and round indentation created with acrylic bur; (C) different shapes were used for fabrication; (D) flasking of the acrylic resins to fabricate sclera

Flasking moulds were prepared by duplicating nine existing ocular prostheses of two different shapes, which were invested in type II gypsum. Definitive prostheses were fabricated using heat‑cured scleral polymer (J-510 Scleral Polymer, Technovent Ltd., South Wales, United Kingdom) combined with A2 and A3 shades of acrylic resin (GC Unifast III, GC Corporation, Tokyo, Japan), mixed according to manufacturer specifications. The resin was packed under a hydraulic press and polymerized in 100°C water for three hours. Following processing, the centre of each prosthesis was marked, and an acrylic bur was used to create a circular indentation measuring 11 mm in diameter and 3 mm in depth (Figures 3A-3C). Five batches, each containing nine moulds, were used (with five prostheses not used), resulting in 40 custom-made ocular prostheses in which irises were constructed according to the following descriptions.

**Figure 4:**
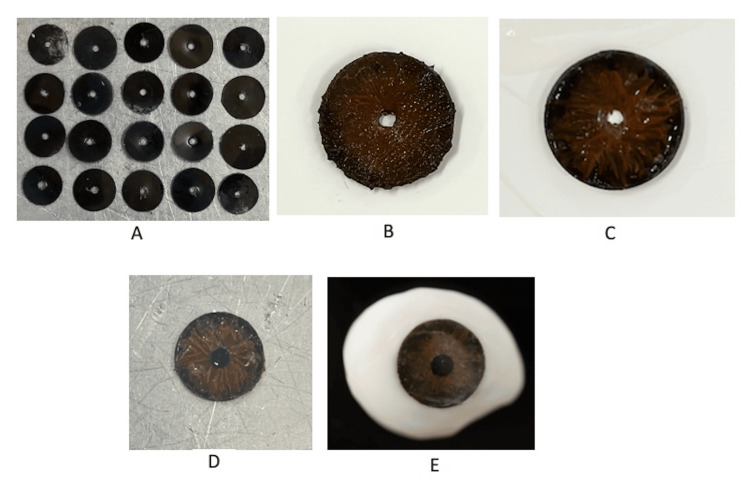
Iris fabrication using oil-painting method: (A) circular 11 mm disks; (B) painted disk; (C) application of monopoly syrup; (D) black bindi placement; (E) completed ocular prosthesis with oil-painted iris

Circular discs of 11 mm diameter were prepared from radiograph phosphor plates. The iris base colour was established by applying a layer of black paint, followed by vertical serrated strokes of brown paint to replicate the natural iris striations once the initial layer had dried. After complete drying, a mixture of monopoly syrup and monomer was applied to seal the painted surface. The pupil was simulated by placing a size 8 black bindi at the centre of each disc. The painted discs were then mounted onto the acrylic sclera and covered with a layer of clear cold-cure acrylic. The prostheses were polymerized in 80°C water at 2.0 atm for 20 minutes. Excess material was removed with an acrylic bur, and the prostheses were polished. The fabrication process resulted in 20 identical oil-painted irises (Figures 4A-4E).

**Figure 5:**
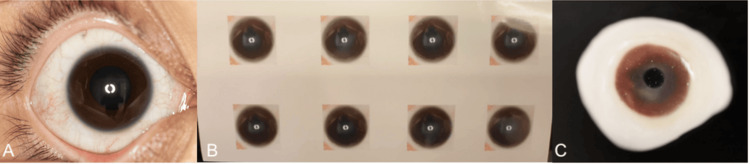
Iris fabrication using photo-printing method: (A) natural human brown iris photograph; (B) photo-printed iris; (C) completed ocular prosthesis with phot-printed iris

The iris of a human subject was photographed in a dark room at a fixed distance of 50 cm using a DSLR camera (Canon EOS RP, Canon Inc., Tokyo, Japan) equipped with a macro lens (Canon EF 100 mm f/2.8L Macro IS USM, Canon Inc., Tokyo, Japan) and ring flash (Macro Ring Lite MR-14EX II, Canon Inc., Tokyo, Japan). The captured image was processed in Adobe Photoshop (Adobe Inc., San Jose, California, USA) and resized to a diameter of 11 mm, then photo‑printed and trimmed to the same dimensions. Printer specifications and resolution parameters (dots per inch) were standardized prior to fabrication, and all images were processed under identical software calibration protocols to ensure consistency in colour reproduction. A protective coating of shellac was applied in two layers, each sprayed from a distance of 15 mm. The pupil was simulated by placing a size 8 black bindi at the centre of the disc. The prepared iris was mounted onto the acrylic sclera and covered with a layer of clear cold-cure acrylic. The prostheses were polymerized in 80°C water at 2.0 atm for 20 minutes. Excess material was removed with an acrylic bur, and the prostheses were polished. The fabrication process resulted in 20 identical photo-printed irises (Figures 5A-5C).

Ten oil‑painted and 10 photo‑printed ocular prostheses were subjected to outdoor weathering for three hours per day (11:00-14:00) over a consecutive two‑week period. Prostheses were placed in a clear acrylic box and positioned to ensure direct sunlight exposure. Rainy and cloudy days were excluded, resulting in 16 days of exposure between 1 and 16 January 2025. Environmental conditions during outdoor exposure were monitored to ensure consistency, with exposure limited to clear weather conditions and standardised daily exposure duration. Ambient light intensity was monitored using a lux meter to maintain uniformity across observation periods.

Before and after exposure, all prostheses were photographed on the same day using a DSLR camera with a macro lens to facilitate before and after images of the ocular prostheses. Camera settings were standardized at ISO 100-300, aperture F1.8-2.8, and shutter speed 1/100-1/250. Photographs were taken at a 1:1 ratio with the lens positioned perpendicular to the sample surface. The room was kept dark to eliminate interference from ambient light, and a matte black sheet was placed behind the prostheses to absorb reflections and maintain focus on the samples. Luminescence was monitored throughout the procedure using a smartphone‑based lux meter (Lux Light Meter; DoggoApps, Lima, Peru).

**Figure 6:**
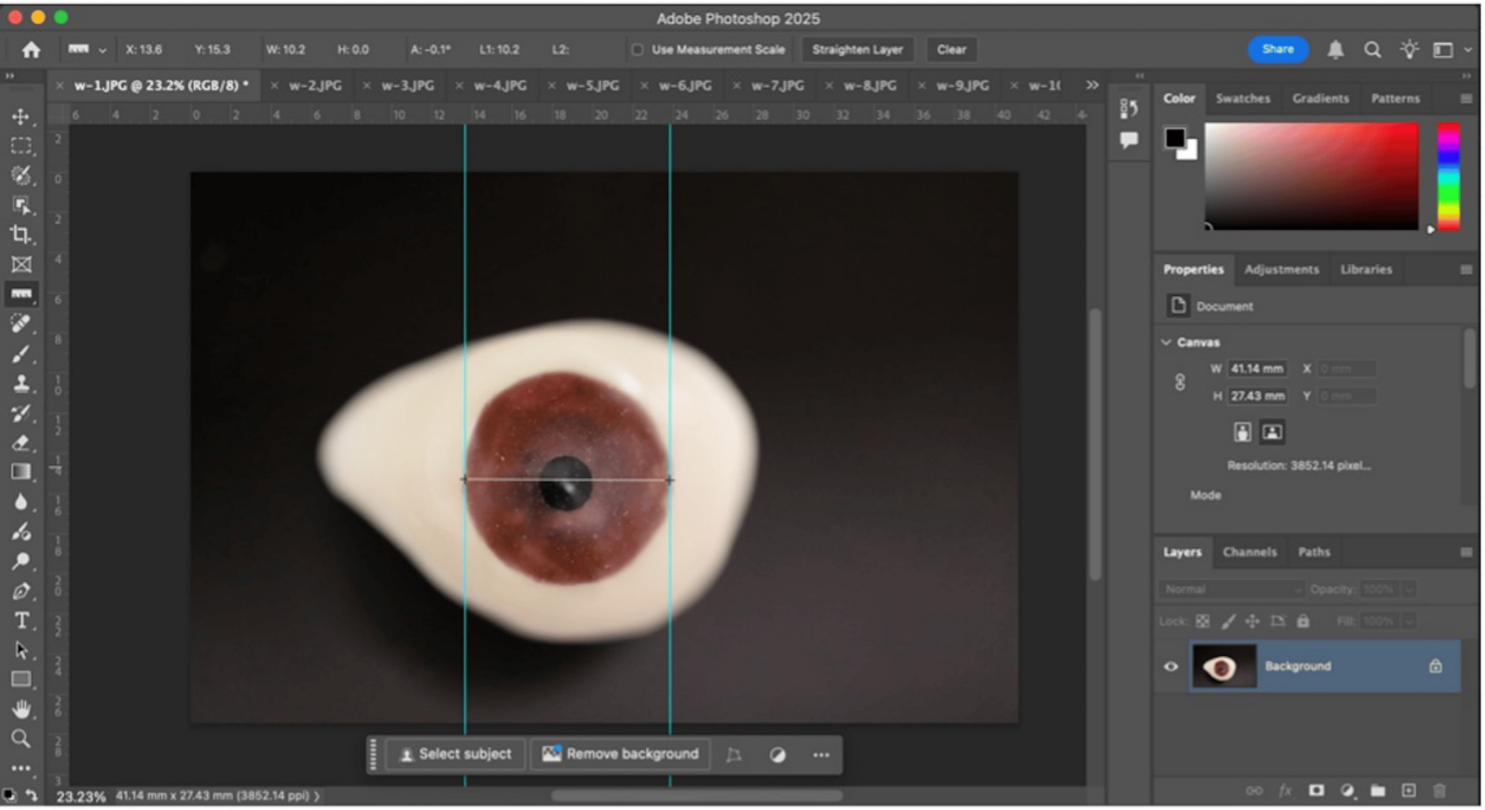
Measurement of iris using Adobe Photoshop

To ensure uniformity in image processing, measurement units in Adobe Photoshop 2025 (Adobe Inc.) were standardized. The Ruler tool in Adobe Photoshop 2025 was used to measure the horizontal diameter of each iris. A line was drawn from edge to edge across the iris, and the L1 length value was recorded from the Options Bar in either pixels or millimetres (Figure 6).

To standardize image resolution, a photograph of an object of known size was captured alongside the irises. The reference object’s length (L1) was measured in pixels using the Photoshop Ruler tool as described previously. Pixels per inch (PPI) were then calculated using the following formula: PPI = Pixels x 25.4/mm.

The Image Size option was selected with Resample turned off, and the calculated PPI value was entered into the Resolution field. All irises were measured in a standardized orientation, with the same PPI calibration applied across all irises to ensure consistency. Measurements were systematically recorded in Microsoft Excel (Microsoft Corp., Redmond, WA, USA).

**Figure 7:**
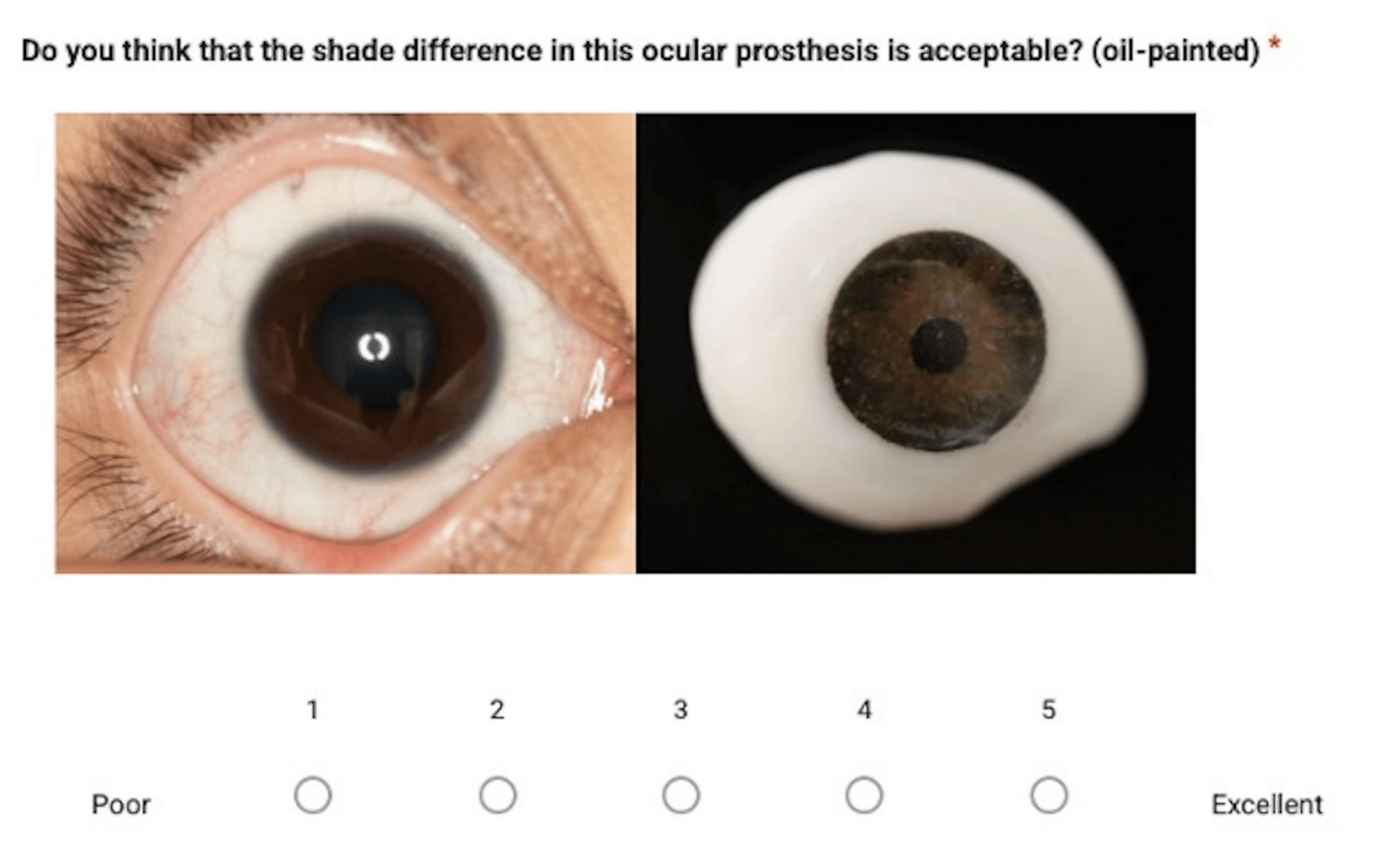
Sample question in the questionnaire

A researcher‑designed questionnaire was used to compare the outcomes of oil-painted and photo-printed prostheses. Subjective acceptability was assessed using this questionnaire, while objective acceptability was determined through quantitative colour (ΔE) and dimensional measurements. The questionnaire employed a five‑point Likert scale and required respondents to rate each item from 1 (‘poor’) to 5 (‘excellent’). Scores of 1 and 2 were classified as not acceptable, a score of 3 was considered neutral, while scores of 4 and 5 were classified as acceptable. Undergraduate dental students were invited to the questionnaire via a survey link administered using Google Forms (Google LLC, Mountain View, California, USA) (Table 1 and Figure 7).

**Table 1:** Questionnaire

Questionnaire item
Q1. Do you think that the shade differences in this particular oil-painted prosthesis are acceptable?
Q2. Do you think that the shade differences in this particular photo-printed prosthesis are acceptable?
Q3. Do you think that the dimension in this particular oil-painted prosthesis is acceptable?
Q4. Do you think that the dimension in this particular photo-printed prosthesis is acceptable?
Q5. Do you think that the aesthetic outcome of this oil-painted ocular prosthesis is acceptable?
Q6. Do you think that the aesthetic outcome of this photo-printed ocular prosthesis is acceptable?

Images were transferred in RAW format and displayed on a computer screen (HP Inc., Palo Alto, California, USA; resolution 1920 × 1080) and uploaded to Adobe Photoshop Lightroom Classic CC (Adobe Systems Inc.) for processing [6]. White balance calibration was performed using the Macbeth colour chart and followed a standardized workflow involving importing images into Lightroom, then using the Developer tool to select the Eyedropper tool and apply the 18% grey reference. The colour chart region was cropped using the Crop Overlay option, and calibration was refined with SpyderCHECKR 24 software (Datacolor Inc., Lawrenceville, New Jersey, USA) by matching the provided colour boxes with the photographed chart. The calibrated image was saved as a preset, which was then applied to subsequent images, with the 18% grey reference re‑selected. Final colour measurements (L, a, b* values) were obtained by placing the cursor on the marked area of the ocular prostheses. The final colour measurement was recorded.

Colour changes in the irises were quantified using the ∆E value. The colour difference (ΔE) was calculated using the CIELAB formula:

$\Delta E = \sqrt{(\Delta L)^2 + (\Delta a)^2 + (\Delta b)^2}$

where ΔL represents changes in lightness, Δa represents red-green variation, and Δb represents yellow-blue variation. This cumulative value reflects perceptible colour change as experienced by the human eye.

The L, a, and b* colour coordinates were extracted from Adobe software, with measurements obtained through the Eyedropper tool. The overall change in colour between case (weathered) and control (non-weathered) groups was quantified using the CIELAB colour difference formula. ∆E was used as the quantitative metric to evaluate perceptible colour differences and represented the overall change in colour as perceived by the human eye, integrating variations in lightness (∆L), red-green axis (∆a), and yellow-blue axis (∆b) within the CIELAB colour space. The individual colour difference components were calculated as follows:

$\Delta L = L^{*}_{\mathrm{sample}} - L^{*}_{\mathrm{standard}}$ $\Delta a = a^{*}_{\mathrm{sample}} - a^{*}_{\mathrm{standard}}$ $\Delta b = b^{*}_{\mathrm{sample}} - b^{*}_{\mathrm{standard}}$

Data analysis was performed using IBM SPSS Statistics for Windows, Version 21 (Released 2012; IBM Corp., Armonk, New York, United States). Normality of data distribution was initially assessed using the Kolmogorov-Smirnov test. Parametric tests were applied when data met assumptions of normality, and non‑parametric tests were used otherwise. Colour change (∆E) was compared across the four groups using one‑way analysis of variance (ANOVA). Post‑hoc comparisons were conducted with the Bonferroni test. Statistical significance was set at p≤0.05.

## Results

The mean colour values across the four experimental groups are shown in Table 2. Photo-printed non-weatheredprostheses (n=10) demonstrated the smallest mean colour value of all other groups. The mean iris sizes across the four experimental groups are shown in Table 3. The photo-printed weathered iris (n=10) demonstrated the smallest mean size compared with the three other groups.

**Table 2:** Colour values for the four experimental groups

Group	Mean	Standard deviation	95% Confidence interval	Range
Oil-painted (weathered) (n=10)	38.9146	3.74271	(36.2373, 41.5920)	11.72
Oil-painted (non-weathered) (n=10)	34.2658	6.05647	(29.9333, 38.5984)	18.54
Photo-printed (weathered) (n=10)	40.3001	7.64568	(34.8307, 45.7695)	21.77
Photo-printed (non-weathered) (n=10)	17.4088	4.89265	(13.9088, 20.9088)	12.73

**Table 3:** Iris size measurements for the four experimental groups

Group	Mean	Standard deviation	95% Confidence interval	Range
Oil-painted (weathered) (n=10)	11.460	0.7199	(10.945, 11.975)	2.3
Oil-painted (non-weathered) (n=10)	11.380	0.8483	(10.773, 11.987)	2.9
Photo-printed (weathered) (n=10)	10.190	0.6343	(9.808, 10.572)	1.9
Photo-printed (non-weathered) (n=10)	11.890	0.7637	(11.344, 12.436)	2.4

The statistical analysis is summarized in Tables 4-11. The normality of the data was first assessed with the Kolmogorov-Smirnov test, which established no statistically significant deviation in normality for the variable. For iris size, the test results showed non-significant p-values (ranging from 0.116 to 0.200), all above 0.05, indicating data for all four groups did not significantly deviate from a normal distribution.

**Table 4:** Tests of normality ^a^ Lilliefors significance correction

Variable	Kolmogorov-Smirnov^a^		Shapiro-Wilk	
	Statistic	Significance	Statistic	Significance
E	0.123	0.130	0.928	0.014

**Table 5:** Tests of normality * Lower bound of the true significance; ^a^ Lilliefors significance correction

Group	Kolmogorov-Smirnov^a^			Shapiro-Wilk		
	Statistic	Df	Sig.	Statistic	Df	Significance
Oil-painted (weathered) (n=10)	0.167	10	0.200*	0.930	10	0.444
Oil-painted (non-weathered) (n=10)	0.238	10	0.116	0.894	10	0.189
Photo-printed (weathered) (n=10)	0.233	10	0.132	0.944	10	0.595
Photo-printed (non-weathered) (n=10)	0.188	10	0.200*	0.941	10	0.563

**Table 6:** Analysis of variance (ANOVA)

E	Sum of squares	Df	Mean square	F	Significance
Between groups	3326.531	3	1108.844	33.328	<0.001>
Within groups	1197.749	36	33.271	-	-
Total	4524.280	39	-	-	-

**Table 7:** Analysis of variance (ANOVA) effect sizes Eta-squared and Epsilon-squared are estimated based on the fixed‑effect model.

E	Effect size	Point estimate	95% CI lower	95% CI upper
	Eta-squared	0.735	0.537	0.806
	Epsilon-squared	0.713	0.499	0.790
	Omega-squared Fixed-effect	0.708	0.492	0.786
	Omega-squared Random-effect	0.447	0.244	0.551

**Table 8:** Analysis of variance (ANOVA) for iris size (mm)

	Sum of squares	Df	Mean square	F	Significance
Between groups	15.926	3	5.309	10.081	<0.001
Within groups	18.958	36	0.527	-	-
Total	34.884	39	-	-	-

**Table 9:** Analysis of variance (ANOVA) effect sizes(a) (a) Eta-squared and Epsilon-squared are estimated based on the fixed‑effect model.

E	Effect size	Point estimate	95% CI lower	95% CI upper
	Eta-squared	0.457	0.171	0.595
	Epsilon-squared	0.411	0.101	0.561
	Omega-squared Fixed-effect	0.405	0.099	0.555
	Omega-squared Random-effect	0.185	0.035	0.293

**Table 10:** Post hoc analysis for the dependent variable E * Mean difference significant at 0.05.

Group (I)	Group (J)	Mean difference (I‑J)	Std. error	Significance	95% confidence interval	
					Lower bound	Lower bound
Oil-painted/weathered (n=10)	Oil-painted/non-weathered	4.64880	2.57957	0.479	-2.5533	11.8509
	Photo-printed/weathered	-1.38542	2.57957	1.000	-8.5875	5.8167
	Photo-printed/non-weathered	21.50584*	2.57957	<0.001>	14.3038	28.7079
Oil-painted/non-weathered (n=10)	Oil-painted/weathered	-4.64880	2.57957	0.479	-11.8509	2.5533
	Photo-printed/weathered	-6.03422	2.57957	0.150	-13.2363	1.1679
	Photo-printed/non-weathered	16.85703*	2.57957	<0.001>	9.6550	24.0591
Photo-printed/weathered (n=10)	Oil-painted/weathered	1.38542	2.57957	1.000	-5.8167	8.5875
	Oil-painted/non-weathered	6.03422	2.57957	0.150	-1.1679	13.2363
	Photo-printed/non-weathered	22.89125*	2.57957	<0.001>	15.6892	30.0933
Photo-printed/non-weathered (n=10)	Oil-painted/weathered	-21.50584*	2.57957	<0.001>	-28.7079	-14.3038
	Oil-painted/non-weathered	-16.85703*	2.57957	<0.001>	-24.0591	-9.6550
	Photo-printed/weathered	-22.89125*	2.57957	<0.001>	-30.0933	-15.6892

**Table 11:** Post hoc analysis for the dependent variable iris size (mm) * Mean difference significant at 0.05.

Group (I)	Group (J)	Mean difference (I‑J)	Std. error	Significance	95% confidence interval	
					Lower bound	Lower bound
Oil-painted/weathered (n=10)	Oil-painted/non-weathered	0.0800	0.3245	0.995	-0.794	0.954
	Photo-printed/weathered	1.2700*	0.3245	0.002	0.396	2.144
	Photo-printed/non-weathered	-0.4300	0.3245	0.553	-1.304	0.444
Oil-painted/non-weathered (n=10)	Oil-painted/weathered	-0.0800	0.3245	0.995	-0.954	0.0794
	Photo-printed/weathered	1.1900*	0.3245	0.004	0.316	2.064
	Photo-printed/non-weathered	-0.5100	0.3245	0.407	-1.384	0.364
Photo-printed/weathered (n=10)	Oil-painted/weathered	-1.2700*	0.3245	0.002	-2.144	-0.396
	Oil-painted/non-weathered	-1.1900*	0.3245	0.004	-2.064	-0.316
	Photo-printed/non-weathered	-1.17000*	0.3245	<0.001	-2.574	-0.826
Photo-printed/non-weathered (n=10)	Oil-painted/weathered	0.4300	0.3245	0.553	-0.444	1.304
	Oil-painted/non-weathered	0.5100	0.3245	0.407	-0.364	1.384
	Photo-printed/weathered	1.7000*	0.3245	<0.001	0.826	2.574

A one-way ANOVA was performed to determine whether values in 40 samples within and between the groups differed when the duration of exposure was kept constant. The result revealed significant differences in colour change values among the 40 samples (F=33.328, p<0.001), leading to rejection of the null hypothesis. The large effect size (η^2^=0.735) indicated that 73.5% of the variance was attributed to group differences, underscoring the substantial impact of the experimental conditions. The ANOVA results also revealed a statistically significant difference in iris size among the four groups (F=10.081, p<0.001). The effect size was large, with eta-squared (η^2^=0.457) suggesting that 45.7% of the variance in iris size was determined by the type of iris.

The photo-printed non-weathered group (n=10) had the lowest mean colour value (17.41), which aligns with significant post hoc differences. Hence, post hoc analysis using the Bonferroni test was performed. The test revealed that colour changes in photo-printed non-weathered irises were significantly greater than those in oil-painted non-weathered irises, oil-painted weathered irises, and photo-printed weathered irises (mean difference). The 95% CI between the photo-printed non-weathered iris group and other groups did not overlap, therefore, reinforcing these findings.

As the ANOVA test revealed a statistically significant difference in the iris size among the experimental groups, a post hoc Tukey’s Honest Significant Difference (HSD) test was performed. The photo-printed weathered iris demonstrated the smallest mean size compared with the three other groups, which aligned with significant post hoc differences. The post hoc HSD test revealed the photo-printed weathered irises were significantly smaller than oil-painted non-weathered irises (ΔM=1.190 mm, 95% CI (0.316, 2.064), p=0.004), photo-printed non-weathered irises (ΔM=1.700 mm, 95% CI (0.826, 2.574), p<0.001), and oil-painted weathered irises (ΔM=1.270 mm, 95% CI (0.396, 2.144), p=0.002). The exclusion of zero from the 95% CI further suggested the reliability of these differences.

A total of 81 respondents completed the questionnaire evaluating the acceptability of oil‑painted and photo‑printed irises (Table 12). For shade difference, oil‑painted irises were rated acceptable by 42 respondents (51.9%), compared with 12 respondents (14.9%) for photo‑printed irises, while photo‑printed irises were more frequently rated as not acceptable (42 respondents, 51.8%) than oil‑painted irises (12 respondents, 14.8%). Regarding dimension, oil‑painted irises received acceptable ratings from 54 respondents (66.7%), whereas only 21 respondents (25.9%) rated photo‑printed irises as acceptable; in contrast, photo‑printed irises were rated as not acceptable by 37 respondents (45.7%). For the overall aesthetic outcome, oil‑painted irises were judged acceptable by 49 respondents (60.6%), compared with 22 respondents (27.1%) for photo‑printed irises. Overall, oil‑painted irises consistently demonstrated higher acceptability ratings across all evaluated domains.

**Table 12:** Summarized questionnaire results (n=81)

Comparison with human iris	Iris type	Not acceptable, n (%)	Neutral, n (%)	Acceptable, n (%)
Shade difference	Oil-painted	12 (14.8)	27 (33.3)	42 (51.9)
	Photo-printed	42 (51.8)	27 (33.3)	12 (14.9)
Dimension	Oil-painted	5 (6.1)	22 (27.2)	54 (66.7)
	Photo-printed	37 (45.7)	23 (28.4)	21 (25.9)
Aesthetic outcome	Oil-painted	14 (17.2)	18 (22.2)	49 (60.6)
	Photo-printed	37 (45.7)	22 (27.2)	22 (27.1)

## Discussion

The findings of this study highlighted that oil-painted irises in ocular prostheses exhibited greater colour and dimensional stability and were judged more acceptable than photo-printed irises in ocular prostheses following exposure to outdoor weathering conditions. These results provide clear evidence that the iris fabrication method significantly influences the performance of the iris component of the ocular prostheses. Therefore, the null hypotheses were rejected.

The greater colour stability observed in oil‑painted irises may be attributed to the inherent properties of traditional pigments and their resistance to photodegradation under ultraviolet and environmental stressors [4]. In contrast, photo‑printed irises rely on inkjet or digital printing technologies, which are more susceptible to fading, pigment migration, and dimensional distortion when exposed to prolonged outdoor weathering [7,8]. These findings align with previous reports highlighting the vulnerability of printed substrates to environmental degradation, while supporting the durability of hand‑applied pigment layers in prosthetic applications [4,9]. The higher acceptability ratings for oil‑painted irises further suggest that their visual qualities more closely replicate the natural human iris, reinforcing the clinical relevance of fabrication method in achieving long‑term aesthetic success [4,9].

The photo-printed non-weathered iris prostheses exhibited the lowest mean colour value in comparison with other groups. As an additive manufacturing process, photo-printing enables precise material deposition layer-by-layer, which may produce distinctive surface characteristics and microstructural variations compared to conventional oil-based techniques [10]. Weathering demonstrated a substantial impact on photo-printed prostheses compared to their oil-based counterparts. This disparity implies that photo-printed materials may respond distinctively to environmental factors, most likely due to their surface characteristics, with several additional variables influencing these outcomes, including the curing process, type of acrylic resin, colouring agents, printing paper, printer model, and resolution (DPI) [7,11].

The photo-printed weathered irises exhibited the smallest mean size, suggesting the material was susceptible to expansion under environmental stress. Photo‑printed irises typically employ dyes layered onto a substrate, and these dyes may be susceptible to ultraviolet degradation from direct sunlight exposure [7,12]. According to Image Permanence Institute’s guide for the preservation of digitally printed images, signs of deterioration may include delamination, yellowing, cracking and other defects, all of which can contribute to an apparent decrease in iris size [13]. In addition, the clear acrylic resin itself may undergo shrinkage due to heat and ultraviolet-induced changes, which could pull the photo-printed layer inwards if it is strongly bonded to the acrylic resin [14]. In contrast, oil-painted irises in both the weathered (case) and non-weathered (control) groups maintained nearly identical mean sizes. This stability is most likely attributable to the inherent properties of oil paint, which contains fewer volatile components and demonstrates greater resistance to shrinkage compared with photo-printed weathered irises. Furthermore, the application of monopoly syrup onto the oil-painted irises as a fixing medium further stabilized the paint [15].

The study was subject to some limitations that should be considered when interpreting the findings. The two-week artificial weathering protocol may not fully replicate long-term real-world degradation processes. The relatively small sample size (n per group) may have reduced the statistical power to detect smaller but potentially meaningful differences. The study only focused on colour change without looking at other clinically relevant material properties such as surface roughness, mechanical strength, or chemical composition changes that could affect prosthesis performance.

Although a standardized measurement protocol was applied, it may not have captured all clinically relevant dimensional changes. The study did not assess the detailed material composition of the paper used for photo-printed irises, which could influence stability under environmental stress. Measurements were taken at a single time point after weathering, limiting insight into longitudinal degradation patterns.

Objective aesthetic evaluations were based on two-dimensional images, which may not fully represent the three-dimensional nature of the ocular prostheses. The subjective nature of aesthetic perception introduces variability in participant ratings, as aesthetic perception varies between individuals. In addition, the use of a single iris colour (brown) and controlled laboratory conditions may limit the generalizability of the findings to broader clinical scenarios involving diverse iris colours and environmental exposures. Complete blinding of investigators was not feasible due to the nature of fabrication and measurement procedures, which may introduce potential observer bias. The results reflect population-level differences only, and in a defined population, as each prosthesis was only measured once rather than longitudinally. These limitations also highlight the need for larger-scale studies incorporating material analysis and longitudinal follow-up to strengthen the findings. While the findings are supported by controlled experimental data, the short‑term in vitro design limits direct extrapolation to long‑term clinical performance, and future longitudinal studies are required to validate these outcomes under real‑world conditions.

## Conclusions

Within the limitations of this in vitro study, oil-painted irises showed significantly greater colour stability and dimensional stability than photo-printed irises following exposure to outdoor weathering conditions. In addition, oil-painted irises were consistently rated as more acceptable in terms of shade, dimension, and overall aesthetic outcome. These findings suggest that the method of iris fabrication has a direct impact on both the durability and aesthetic performance of ocular prostheses. Conventional oil-painting techniques remain a more predictable approach for achieving stable and clinically acceptable results, particularly in environments where prostheses may be exposed to environmental stressors. While photo-printing offers potential advantages in standardization and efficiency, its susceptibility to colour change and dimensional alteration under weathering conditions limits its current clinical reliability. Further research is required to improve digital fabrication materials and techniques and to evaluate long-term outcomes under clinical conditions.
